# Full real-space analysis of a dodecagonal quasicrystal

**DOI:** 10.1107/S2053273319000056

**Published:** 2019-02-28

**Authors:** Sebastian Schenk, Eva Maria Zollner, Oliver Krahn, Berit Schreck, René Hammer, Stefan Förster, Wolf Widdra

**Affiliations:** a Institute of Physics, Martin-Luther-Universität Halle-Wittenberg, Halle, Germany; b Max-Planck-Institut für Mikrostrukturphysik, Halle, Germany

**Keywords:** 2D oxide quasicrystal, BaTiO_3_ on Pt(111), dodecagonal tiling, statistical analysis, scanning tunnelling microscopy

## Abstract

The atomically resolved real-space structure of a long-range-ordered dodecagonal quasicrystal is determined based on scanning tunnelling microscopy. For the BaTiO_3_-derived oxide quasicrystal which spontaneously forms on a Pt(111) surface, 8100 atomic positions have been determined and are compared with an ideal Niizeki–Gähler tiling. Although the Niizeki–Gähler tiling has a complex three-element structure, the abundance of the triangle, square and rhomb tiling elements in the experimental data closely resembles the ideal frequencies. Similarly, the frequencies of all possible next-neighbour tiling combinations are, within the experimental uncertainty, identical to the ideal tiling. The angular and orientational distributions of all individual tiling elements show the characteristics of the dodecagonal quasicrystal. In contrast, the analysis of the orientation of characteristic and more complex tiling combinations indicates the partial decomposition of the quasicrystal into small patches with locally reduced symmetry. These, however, preserve the long-range quasicrystal coherence. The symmetry reduction from dodecagonal to sixfold is assigned to local interaction with the threefold substrate. It leads to atomic flips which preserve the number of quasicrystal tiling elements.

## Abstract 2

Analysis of a dodecagonal quasicrystal based on 8100 vertices from atomically resolved scanning tunnelling microscopy has been carried out. A detailed frequency and orientational analysis is presented for the triangle–square–rhomb tiling of a BaTiO_3_-derived quasicrystal.

## 1. Introduction   

A real-space analysis of quasicrystal (QC) tilings is in general a very difficult task. For icosahedral QCs, that are aperiodic in all three dimensions, averaging methods like high-resolution transmission electron microscopy (HRTEM) cannot be applied. Instead, the information on the QC tiling has typically been obtained from atomically resolved scanning tunnelling microscopy (STM) measurements (Cai *et al.*, 2002 ▸; Papadopolos *et al.*, 2008 ▸; McGrath *et al.*, 2010 ▸). However, systematic statistical studies of icosahedral tilings based on atomic vertex positions have not been reported so far. Dodecagonal QCs exhibit aperiodic order within the dodeca­gonal plane, but periodic order in the perpendicular direction. This reduced complexity allows the averaging along atomic rows perpendicular to the dodecagonal plane which has led to dodecagonal structure determination in intermetallic alloy QCs (Ishimasa *et al.*, 1985 ▸, 2015 ▸; Chen *et al.*, 1988 ▸; Krumeich *et al.*, 1998 ▸; Iwami & Ishimasa, 2015 ▸). For an Mn–Cr–Ni–Si alloy, Ishimasa *et al.* recently pushed the limit of QC tiling analysis to the 10 nm range using HRTEM (Ishimasa *et al.*, 2015 ▸). In their work QC domains of roughly 2000 vertices have been analysed in physical and phason space. Besides intermetallic systems, dodecagonal structures are also observed in soft matter (Zeng *et al.*, 2004 ▸; Hayashida *et al.*, 2007 ▸; Talapin *et al.*, 2009 ▸; Steurer, 2012 ▸; Chanpuriya *et al.*, 2016 ▸; Fischer *et al.*, 2011 ▸; Iacovella *et al.*, 2011 ▸; Engel & Trebin, 2007 ▸; Dotera *et al.*, 2014 ▸) as well as in ultrathin 2D adlayers on ideal metal surfaces (Förster *et al.*, 2013 ▸; Urgel *et al.*, 2016 ▸; Paßens *et al.*, 2017 ▸).

Here we present a large-area real-space analysis for the 2013-discovered oxide quasicrystal (OQC), which is derived from an ultrathin BaTiO_3_ layer on a single-crystal Pt(111) substrate (Förster *et al.*, 2013 ▸). Its atomic structure is imaged directly by means of scanning tunnelling microscopy (STM). This technique is used for the first time to analyse a QC on an atom-by-atom basis and to address local defects as well as structural coherence on a 50 nm length scale. 8100 atomic positions within a dodecagonal OQC are analysed and compared with high-resolution electron diffraction from the same structure.

## 2. The dodecagonal Niizeki–Gähler tiling   

**Figure 1:**
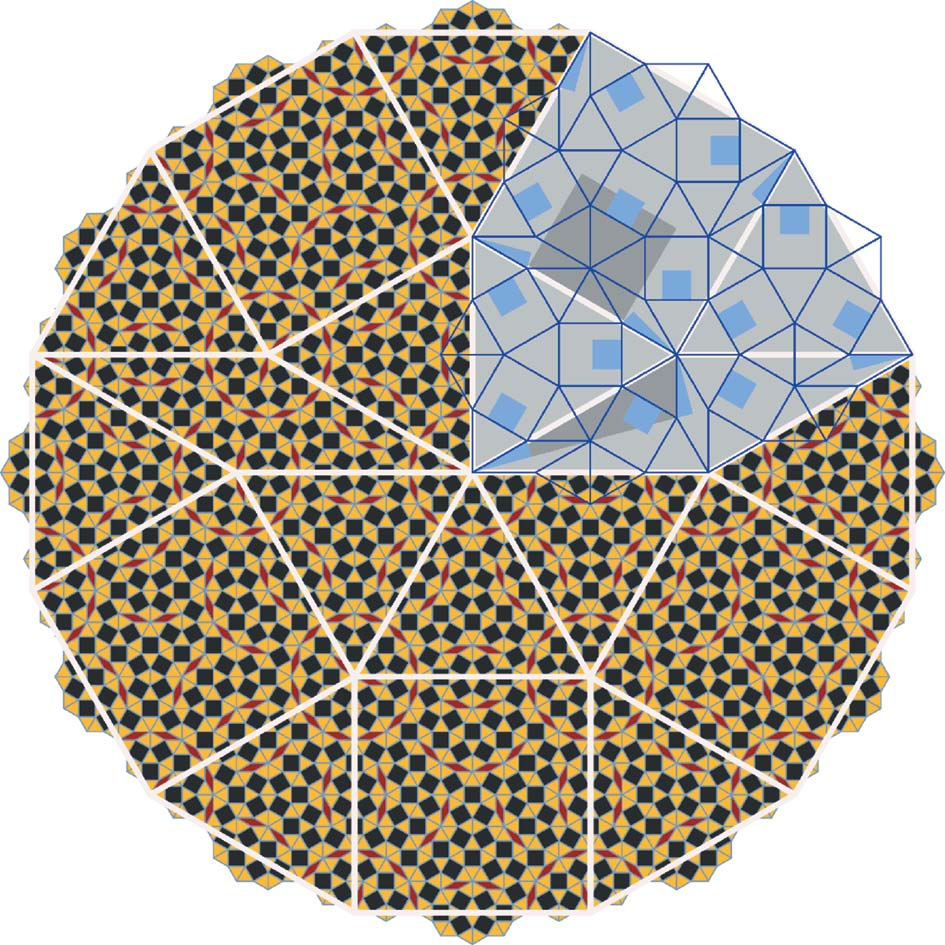
Niizeki–Gähler tiling on different length scales as generated from recursion, emphasizing its self-similarity. The white lines represent a first-generation tiling. In the upper right part the substitution rule for the three elements is given. The symmetry of rhombs and squares is reduced as indicated by the shaded areas. The blue lines represent the tiling deflated once, which scales with 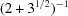. The smallest scale tiling, indicated by black squares, golden triangles and red rhombs, results from a second deflation.

2D OQCs as derived from BaTiO_3_ and SrTiO_3_ on the Pt(111) surface are constructed from three tiling elements of common edge length, namely equilateral triangles, squares and rhombs inclining 30° and 150° angles. Using these elements an ideal dodecagonal tiling can be constructed as introduced independently by Niizeki & Mitani (1987 ▸) and Gähler (1988 ▸). We will refer to this tiling as Niizeki–Gähler tiling (NGT). The NGT exhibits a characteristic higher-order building block, which is a dodecagon, that consists of 12 triangles, five squares and two rhombs as drawn in white in Fig. 1 ▸.

The NGT can be generated by recursion (Liao *et al.*, 2013 ▸) starting from one of the elementary tiling elements or, as shown in Fig. 1 ▸, from the characteristic dodecagon. In a single deflation step, each triangle is replaced by seven triangles and three squares of smaller length. Each square is replaced by 16 triangles, five squares and four rhombs, whereas each rhomb is replaced by eight triangles, two squares and three rhombs. These substitutions give rise to the blue tiling in the upper right of Fig. 1 ▸. According to the recursion rule, the symmetry of squares and rhombs is reduced. The squares have only mirror symmetry due to their decoration with rhombs in the next generation of the tiling. This reduced symmetry is emphasized by the shaded grey and blue areas in Fig. 1 ▸. The presence of squares inside the rhombs upon recursion likewise lowers the symmetry of the rhombs to C_1_.

The substitution can be expressed mathematically using a deflation matrix *T*: 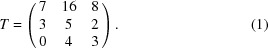
In this representation of *T* the rows of the matrix are assigned to the number of triangle, square and rhomb tiles, respectively, and thus every vector operation on *T* depends on this representation. The eigensystem solution of *T* reveals two important properties of the NGT. Firstly, one can derive the scaling factor of self-similarity in the NGT of (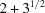) from the square root of the eigenvalue. Secondly, the corresponding eigenvector of the deflation matrix *T*

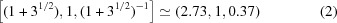
represents the tiling element ratio in the NGT: the numbers of triangles relative to the numbers of squares and of rhombs. For a finite tiling from any start configuration, the number of tiling elements will converge to these values upon multiple deflation iterations. By deflating the white dodecagon in Fig. 1 ▸ twice, one derives a tiling consisting of 2708 vertices, which contains 2640 triangles (gold), 953 squares (black) and 338 rhombs (red). The corresponding tiling frequency of 2.75:1:0.38 already closely resembles the ideal value.

## 3. Experimental   

Ultrathin BaTiO_3_ films were grown on Pt(111) by radiofrequency-assisted magnetron sputter deposition as reported elsewhere (Förster & Widdra, 2010 ▸). The OQC develops upon annealing the BaTiO_3_ films at temperatures above 1150 K in ultrahigh vacuum (UHV) (Förster *et al.*, 2013 ▸). The long-range order has been confirmed by low-energy electron diffraction. For low-temperature STM measurements the sample has been transferred into a home-built STM chamber using an UHV suitcase. The data have been recorded at 77 K.

For conducting the statistical analysis of the length and angular distributions, the STM images have been corrected for piezo-scanner creep and thermal drift in the scanning tunnelling microscope. The background-subtracted data have been corrected by using a plugin to the opensource software *ImageJ* (Schneider *et al.*, 2012 ▸) developed by Michael Schmid (TU Vienna). This plugin applies higher-order non-linear corrections to remove the creep-induced distortions from the STM data by maximizing the intensity of selected spots in the Fourier transform of the image. Subsequently, linear corrections have been applied using *Gwyddion* (Nečas & Klapetek, 2012 ▸). The final STM image has been scaled to meet an average next-neighbour distance of 6.85 Å. This length has been previously evaluated from low-energy electron diffraction (Förster *et al.*, 2013 ▸). The further analysis was done with the help of the image and mesh processing capabilities of *Mathematica* (Wolfram Research).

## 4. Statistical analysis   

**Figure 2:**
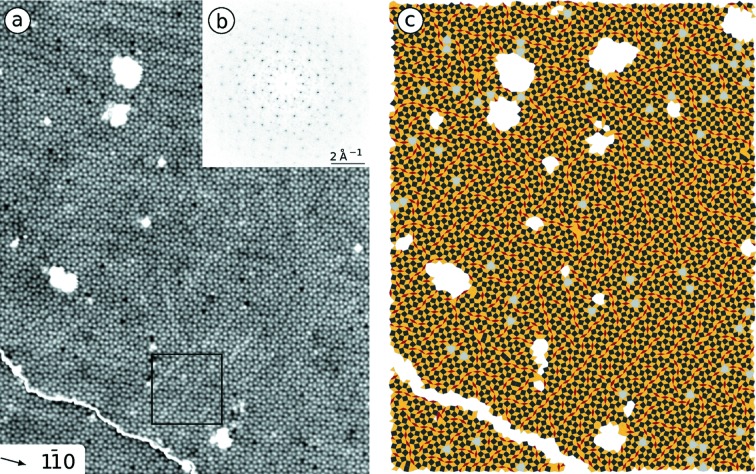
(*a*) STM measurement of BaTiO_3_-derived OQC on Pt(111) showing the sublattice of the Ti atoms. The region marked in black is discussed in more detail in Fig. 8 ▸. (*b*) Fourier transform of the atomic positions extracted from (*a*). (*c*) The OQC tiling as extracted from the atomic coordinates showing triangles (gold), squares (black), rhombs (red) and shields (grey). (*a*) 52 × 67 nm, 15 pm, −1 V.

Fig. 2 ▸(*a*) shows an atomically resolved STM image of the BaTiO_3_-derived OQC. Two terraces are present in this region separated by a monoatomic step [white line in the lower left part of Fig. 2 ▸(*a*)]. The contrast has been adjusted to make the detailed atomic structure of each terrace visible. A purely background-subtracted version of this image is available in the supporting information. The bright protrusions in the STM image arise from the Ti grid of the OQC as previously demonstrated for the sigma phase approximant (Förster *et al.*, 2016 ▸).

The few large white features on the upper terrace are adsorbates that might decorate defects. For all Ti atoms, the atomic coordinates have been determined by fitting the protrusion with 2D Gaussian profiles. From these coordinates the Fourier transform (FT) has been calculated as depicted in Fig. 2 ▸(*b*). The FT shows a pronounced long-range order in the OQC tiling due to the presence of a large number of high-order reflections. Based on the atomic positions, the OQC tiling is extracted as shown in Fig. 2 ▸(*c*). The structure is determined by triangles, squares and rhombs, but also a small number of shield elements can be recognized [grey in Fig. 2 ▸(*c*)]. The latter element results from one missing Ti atom in the structure, which would otherwise be filled by two triangles, one square and one rhomb.

The tiling elements in Fig. 2 ▸(*c*) sum up to 7773 triangles, 2806 squares and 981 rhombs. These numbers correspond to a ratio of 2.77:1:0.35, which is close to that of the ideal NGT. If one additionally takes the 57 shield defects into account, which equal 114 triangles, 57 squares and 57 rhombs, the tiling element ratio will change to 2.75:1:0.36. This even more closely approaches the NGT ratio of 2.73:1:0.37.

**Figure 3:**
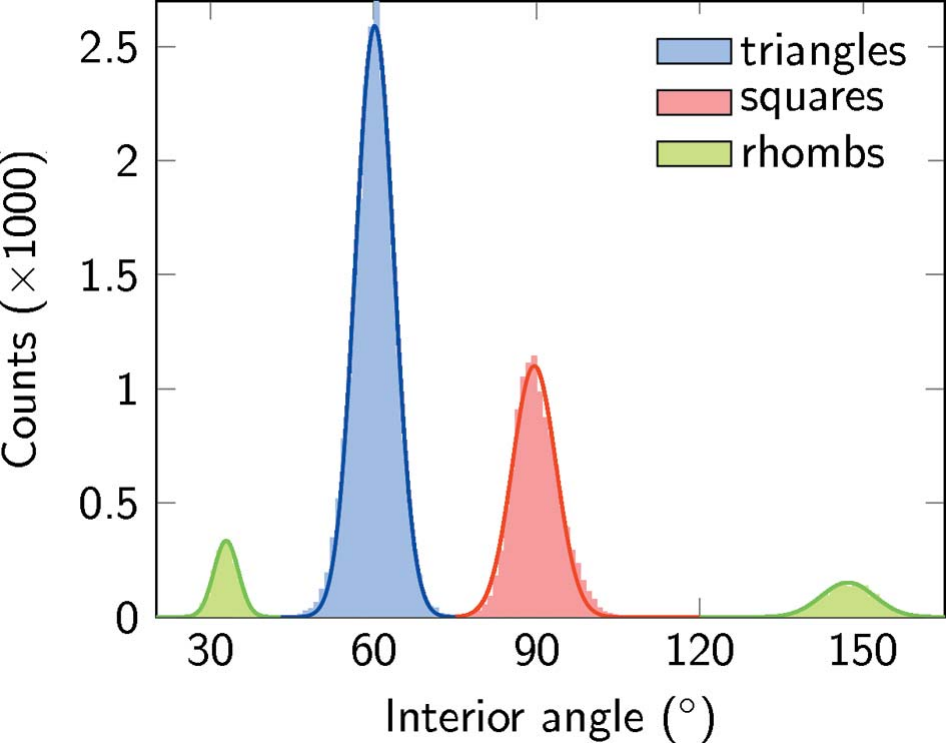
Distribution of interior angles in the triangles, squares and rhombs as derived from the drift-corrected STM data of the OQC tiling in Fig. 2 ▸.

Besides counting the tiling elements, an analysis of the angular distribution of the Ti neighbours around each vertex has been conducted. This distribution is plotted in Fig. 3 ▸.

It gives a statistical measure of the interior angles of triangles, squares and rhombs. The angular distribution of the interior angles for triangles is well described by a Gaussian centred at 60.1° with a full width at half-maximum (FWHM) of 8.3°. For squares, the distribution is asymmetrically broadened to higher angles. From fitting with a Gaussian, the maximum is found at 89.2° and the FWHM is 9.4°. The interior angles of the rhombs strongly deviate from their expected values of 30° and 150°. Instead, two Gaussians can be fitted at 32.7° and 147.3°. Their widths have been determined to 5.2° and 11.5°, respectively. The angular variations as expressed by the broadening of all distributions reflect local distortions of all elements. In the case of triangles and squares, the distortions are balanced. However, the shift of the maximum in the angular distribution of rhombs reflects a systematic deformation. The shift by 2.7° corresponds to a change in the aspect ratio of the diagonals of 9% when assuming constant edge lengths.

**Figure 4:**
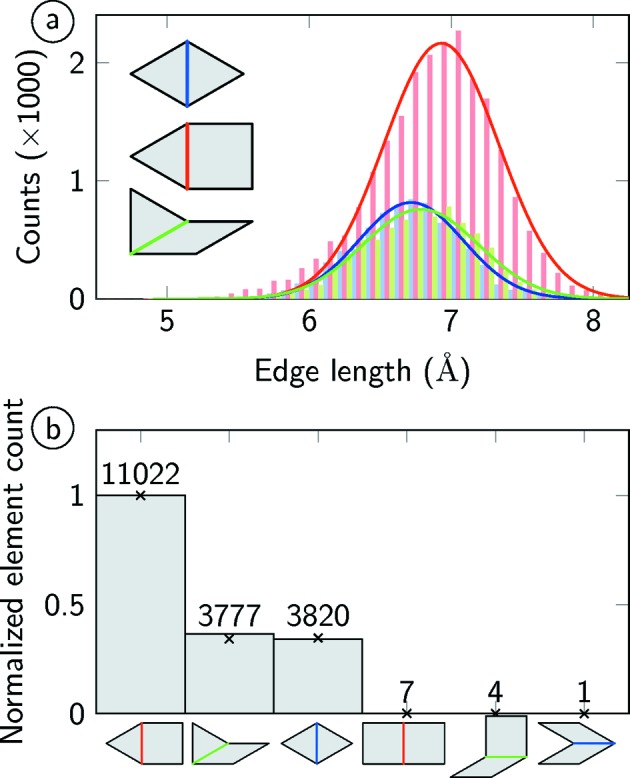
(*a*) Side length distribution of the OQC tiling elements as derived from Fig. 2 ▸. The histograms show the lengths of edges shared between adjacent triangles, between triangles and squares, and between triangles and rhombs in classes of 0.1 Å. The representation in classes causes an off-centring of blue and green bars. (*b*) Number of neighbouring tiling combinations: crosses and absolute numbers mark the experimental values, whereas the grey bar chart indicates the ideal NGT values.

The distributions of side lengths which are shown in Fig. 4 ▸(*a*) for three combinations of neighbouring tiling elements also reveal small deviations from the ideal tiling.

From fitting the histograms with Gaussian distributions, average side lengths of 6.95, 6.72 and 6.79 Å are determined for the combination of triangles with squares, triangles and rhombs, respectively. With an FWHM of 0.95 Å these distributions are again quite broad. The average triangle–square side length is slightly longer than the others. However, its distribution also contains a few short distances around 5.7 Å, which are not covered by the Gaussian. In total, these variations are well within the scattering of the data.

Neighbouring tiling elements in an ideal NGT contain only a negligible number of rhomb–rhomb, rhomb–square or square–square contacts. The only relevant combinations are triangle–square, triangle–rhomb and triangle–triangle, where the first of these has the highest abundance as indicated in Fig. 4 ▸(*b*) as a grey bar chart. The experimentally determined frequencies for the OQC are marked by crosses for all six tiling combinations. The experimental values are in perfect agreement with those of an ideal NGT. This includes the correct relative frequency of the triangle–square, triangle–triangle and triangle–rhomb edges as well as the absence of rhomb–rhomb, rhomb–square and square–square edges.

**Figure 5:**
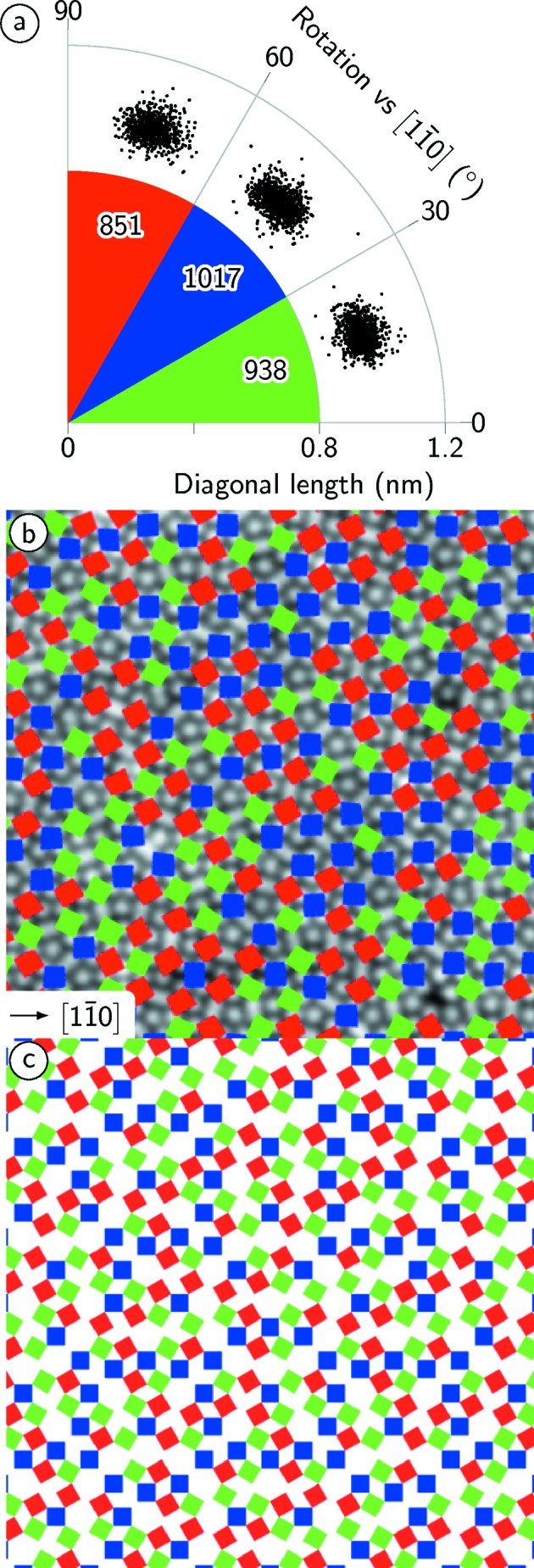
(*a*) The distribution of squares from Fig. 2 ▸. The length of the diagonals is determined and their rotational distribution relative to the 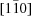 substrate direction is given in classes of 30°. (*b*) Superposition of differently oriented squares to a cut of Fig. 2 ▸(*a*) for comparison with (*c*) the ideal NGT.

Besides the tiling statistics, the rotational alignment of the different elements with respect to the 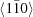 Pt(111) substrate directions has been analysed in detail. Figs. 5 ▸, 6 ▸ and 7 ▸ show the orientation of squares, rhombs and the characteristic dodeca­gons, respectively. In all cases the results taken from the STM image are compared with the arrangement of these units in an ideal NGT.

The detailed analysis for the squares is given in Fig. 5 ▸(*a*) in which their orientational distribution and the variations in the length of their diagonals are plotted. Within each type of square, a spreading of the orientation and of the lengths is observed, which might emphasize distortions within the tiling. However, the distribution reveals no correlation between the fluctuations in length and bond direction. From diffraction, the orientation of the OQC with respect to the underlying substrate is known (Förster *et al.*, 2013 ▸). It turns out that the diagonals of the squares are rotated by 15° with respect to the atomic rows of Pt(111) along the 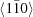 directions. Correspondingly, each square is oriented with one edge along this high-symmetry direction.

Fig. 5 ▸(*a*) reveals a 10% variation in the frequency of differently oriented squares. In the ideal NGT the orientation of squares is homogeneously distributed as shown in Fig. 5 ▸(*c*) with the same frequency in all three rotations. In contrast, the OQC data in Fig. 5 ▸(*b*) show an arrangement where locally one orientation is suppressed. In the ideal NGT and in the measured data, no square shares a corner with a second one of the same orientation. In the ideal NGT a square is never connected to more than two squares of common orientations, and up to four neighbours of identical orientation are found in the real OQC tiling.

**Figure 6:**
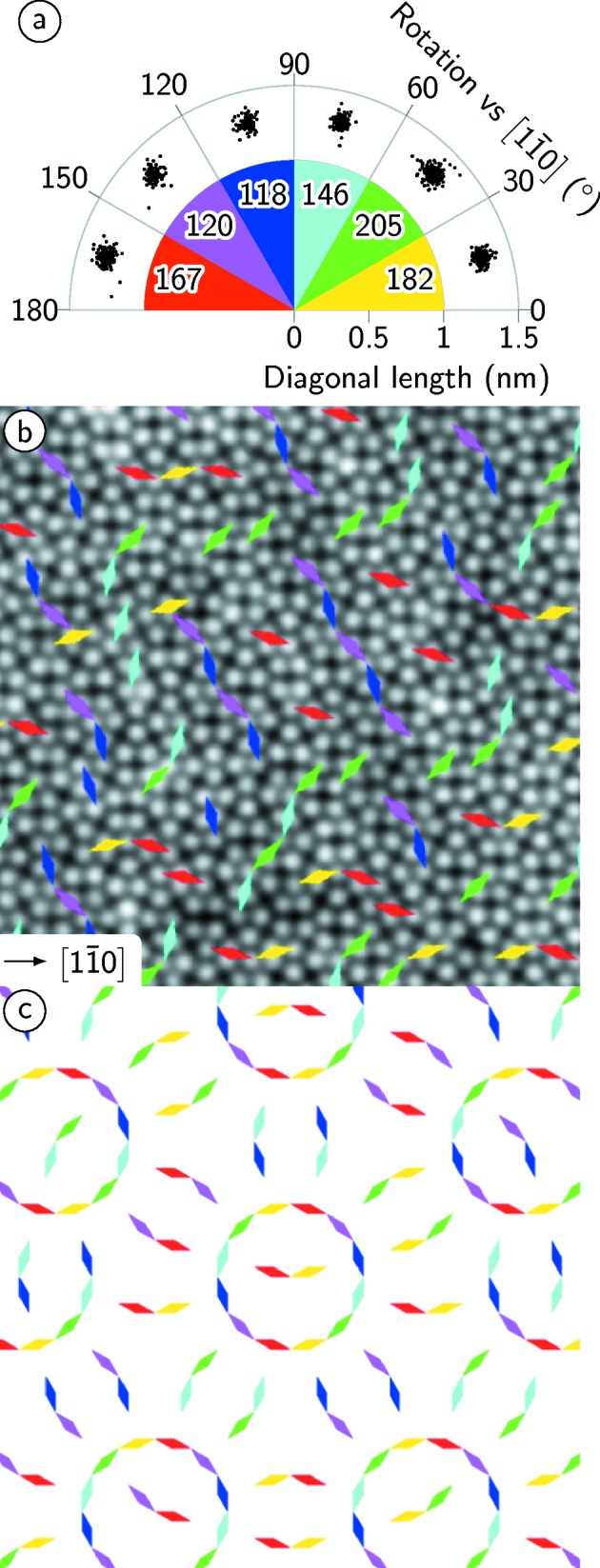
(*a*) Raw data of the rotational distribution of rhombs from Fig. 2 ▸. The frequency of rhomb orientations taken from their long axes in classes of 30° relative to the 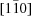 direction of Pt(111) is given. (*b*) Superposition of differently oriented rhombs to a cut of Fig. 2 ▸(*a*) for comparison with (*c*) the ideal NGT.

The rhombs occur in six different orientations every 30°. Their distribution has been determined from their long axis which is shown as raw data in Fig. 6 ▸(*a*). The variations of lengths and bond angles are similar to the case of squares discussed before.

Note that the frequency of the six different orientations as given by the numbers in Fig. 6 ▸(*a*) reveals deviations up to 30% from the mean value. By comparing the local orientation of rhombs within the real OQC data in Fig. 6 ▸(*b*) with that of the ideal NGT (Fig. 6 ▸ *c*), three major differences can be recognized. Firstly, in the OQC isolated rhombs are present, which is not the case in the ideal NGT. Secondly, the rhombs in the OQC tiling tend to arrange in lines with two edges parallel to the 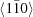 directions. Along these lines their long diagonals are alternately oriented in ±150°. In the ideal NGT, those chains do not occur; instead the rhombs tend to form circles or occur in isolated pairs. Thirdly, the distribution of rhombs within the ideal NGT is equal in all directions for symmetry reasons.

**Figure 7:**
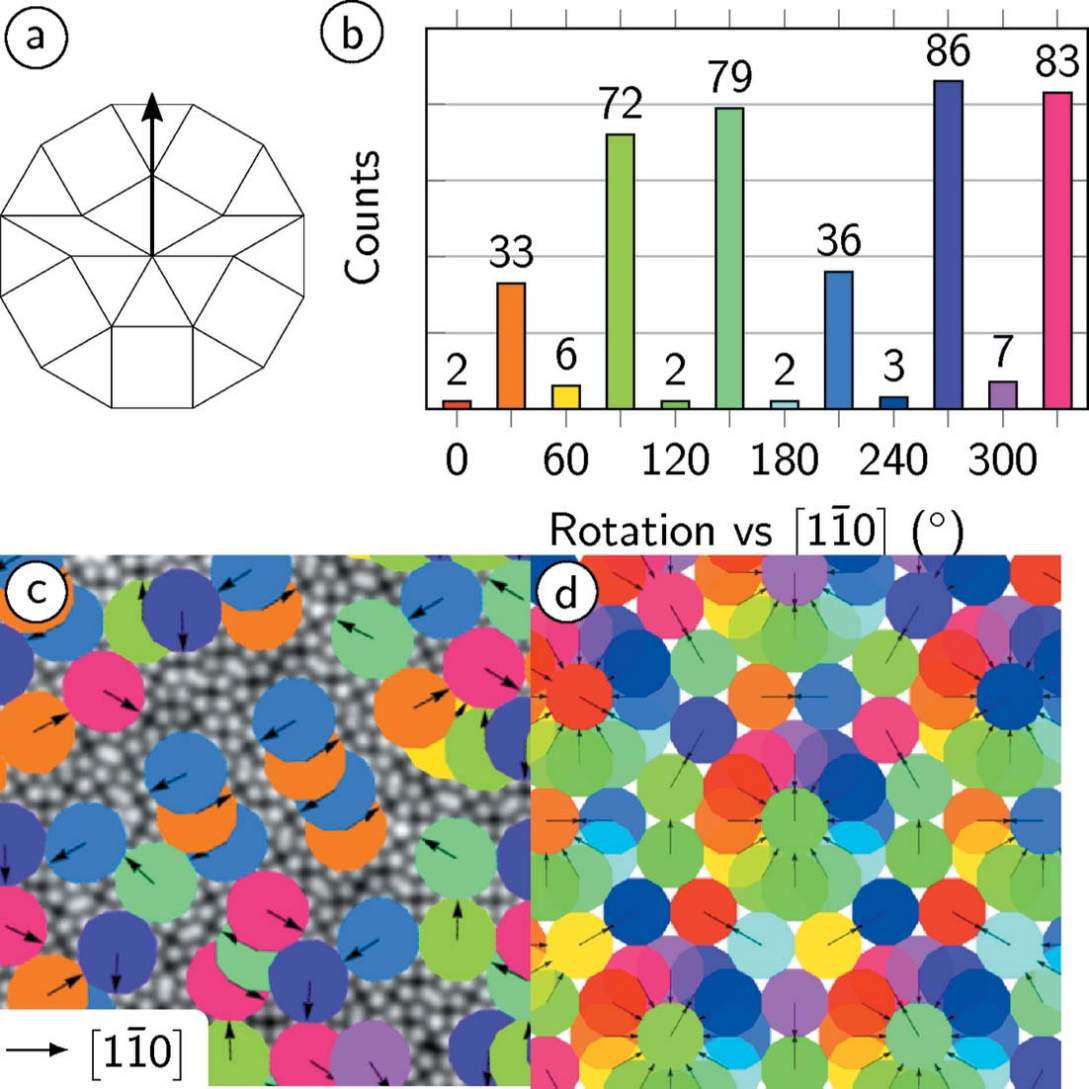
(*a*) Definition of the orientation in the characteristic dodecagon of the NGT. (*b*) Frequency of dodecagon orientations in the OQC in classes of 30° relative to the 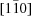 direction of Pt(111). (*c*) Superposition of differently oriented dodecagons to the measured STM data for comparison with (*d*) the ideal NGT.

Finally, the orientation of the characteristic dodecagons of the NGT tiling has been evaluated. We define the orientation of the dodecagon based on its mirror symmetry axis with a direction given as shown schematically in Fig. 7 ▸(*a*). The symmetry of the dodecagon allows 12 orientations.

The histogram of Fig. 7 ▸(*b*) shows the orientational distribution of dodecagons in the OQC and reveals clearly a preference for six out of the 12 directions. Almost all dodeca­gons are found under 30° rotations against the 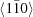 substrate directions. Two orientations at 30° and 210° relative to the 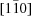 direction occur only half as often in the OQC tiling of Fig. 2 ▸(*a*) as compared with 90°, 120°, 270° and 330°, which emphasizes a correlation of dodecagons with opposite orientations. This becomes more obvious when superimposing the dodecagons to the STM data as shown in Fig. 7 ▸(*c*). The lines that have been recognized in the distribution of rhombs are a consequence of an overlapping of adjacent dodecagons of opposite orientations. This is in strong contrast to the cluster distribution in the ideal NGT, which must again be equally balanced along every 30° shown in Fig. 7 ▸(*d*).

## 5. Discussion   

In the previous section we have presented a detailed statistical analysis for a large area of the dodecagonal BaTiO_3_-derived OQC on Pt(111). The structure has only a very small number of defects and consists mainly of triangle, square and rhomb configurations. Their frequency and rotational orientation are very close to those of the NGT. In addition, the frequencies of the six possible nearest-neighbour configurations, as dominated by triangle–triangle, triangle–square and triangle–rhomb configurations, match perfectly those of the NGT.

However, a number of deviations from the ideal NGT are also reported here. Variations of the average side lengths of the three tiling elements by roughly ±2% around the value of 6.85 Å have been observed. The average spreading of these side lengths is in the order of ±0.5 Å, which is quite substantial. From evaluating the angles between adjacent edges a stretching along the short diagonal of the rhombs by 9% is expected. These deviations from an ideal geometry most likely relate to an adaptation to preferential adsorption sites on the atomic level. These are hard to determine precisely, since only the Ti grid of the BaTiO_3_-derived 2D structure is imaged in STM. The information about the positions of Ba and O atoms is still lacking.

**Figure 8:**
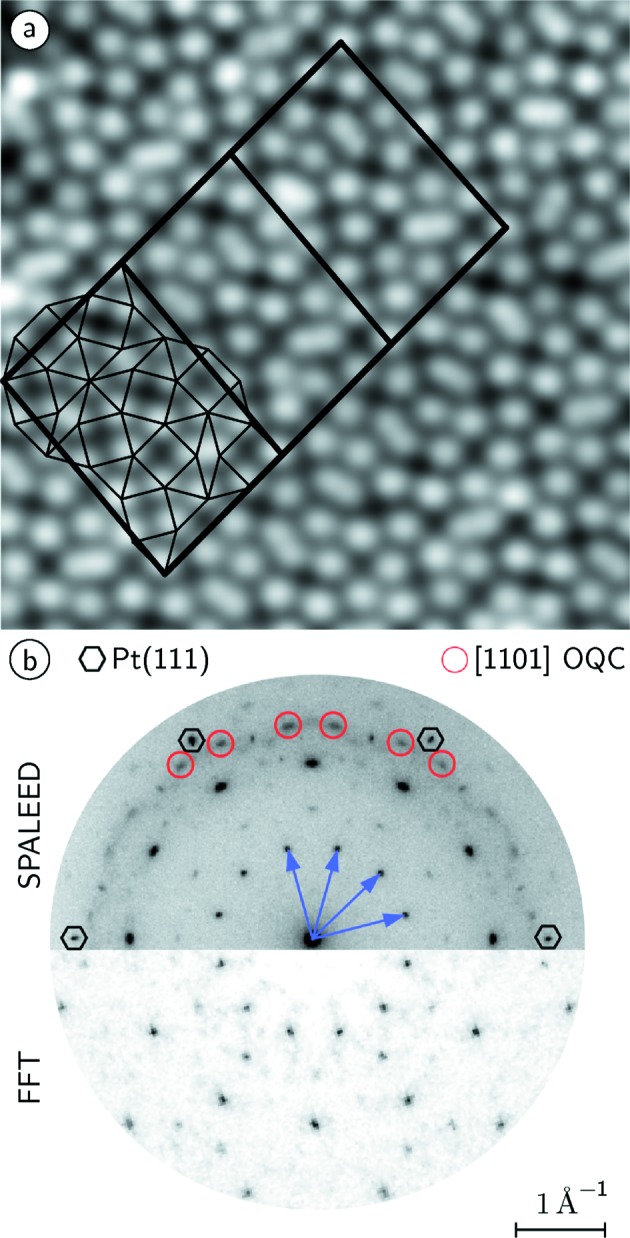
(*a)* Close-up STM image of the BaTiO_3_-derived OQC tiling taken within the black square of Fig. 2 ▸(*a*). Locally, patches of an approximant unit cell can be identified, which consists of 36 tiling elements. (*b*) SPALEED image of the OQC in comparison with the FT from Fig. 2 ▸(*b*).

In addition, a clear sixfold signature is found in the rotational distribution of dodecagons in the OQC tiling. This symmetry reduction is accompanied by the formation of rows of dodecagons in opposite orientations as seen in Fig. 7 ▸(*c*). As a consequence, the circular arrangements of rhombs within the NGT are transformed into a row-like structure. The detailed analysis of the local OQC tiling along these rows identifies individual unit cells of a giant approximant structure that was recently reported for the SrTiO_3_–Pt system (Schenk *et al.*, 2017 ▸). Fig. 8 ▸(*a*) shows a close-up of the STM image of Fig. 2 ▸(*a*). In this area three approximant unit cells are marked by black lines. Their short unit-cell vector is aligned parallel to the 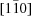 direction. Additionally, the 36 tiling elements within the approximant unit cell are indicated. Interestingly, the unit cell includes one of the characteristic dodecagons discussed in Fig. 1 ▸. Whereas the unit cell is a motif of the ideal NGT, the periodic repetition is not. Therefore, a larger periodic repetition of this unit cell would correspond to an approximant domain embedded in the OQC. In the area given in Fig. 8 ▸(*a*), this motif is repeated a few times along the short unit-cell vector, which produces the row-like structure of the rhombs and oppositely oriented dodecagons. Therefore, it is important to compare the tiling statistics for the ideal NGT, the ideal approximant structure and the experimentally observed structure. The triangle:square:rhomb ratios for the NGT are 2.73:1:0.37, whereas the approximant ratios are 2.66:1:0.33. In the approximant are fewer triangles and fewer rhombs in comparison with the NGT. The experimentally observed ratios are 2.75:1:0.36 and are very close to those of the NGT. In fact, the small deviations cannot be explained by approximant domains within the NGT, since the frequency of triangles is slightly higher in the experiment as compared with the NGT. Note that the triangle frequency is lower in the approximant. Therefore, we conclude that the approximant patches are in the limit of vanishing domain size. A second proof of the very local nature of the symmetry reduction to a sixfold structure comes from the comparison of the FT of the atomic grid as determined in the STM image and the global diffraction pattern obtained by spot-profile analysis of low-energy electron diffraction (SPALEED) as shown in Fig. 8 ▸(*b*). Whereas the FT has been calculated from 3500 nm^2^, the diffraction data are collected from an area of 5 mm × 5 mm. The major difference between both patterns is the intensities of the 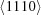 higher-order spots, marked by red circles in Fig. 8 ▸(*b*). Their intensity distribution implies a local symmetry reduction to sixfold in the FT of this STM image. However, the real diffraction data in Fig. 8 ▸(*b*) show clearly the long-range coherence for a dodecagonal quasicrystal.

On the one hand, the giant approximant structure is very *locally* present in different orientations. On the other hand, the long-range dodecagonal order and coherence are *globally* maintained despite the observed local fluctuations. One scenario which can combine both aspects arises from an initially very well developed QC at growth temperatures. At these higher temperatures, the QC can be additionally stabilized by the enhanced QC entropy. Upon cooling, the interaction with the substrate favours specific adsorption sites. The QC structure transforms locally into approximant-like geometries if these structures match the substrate lattice. However, these changes can occur only without significant mass transport, *e.g.* by local site changes. In fact, such local changes of atomic positions in a QC that leave the number of tiling elements unchanged, as we observe here, are known as phason flips.

## 6. Conclusion   

An in-depth statistical analysis of the dodecagonal tiling of the BaTiO_3_-derived oxide quasicrystal on the Pt(111) substrate is presented. The tiling ratio which is determined from almost 11 500 elements created from 8100 vertex positions unambiguously identifies the ideal NGT formed from equilateral triangles, squares and rhombs as the host structure of the BaTiO_3_-derived OQC. For different elements of the OQC tiling strong distortions are found, both in their interior angles and their edge lengths, which most likely occur due to local preferences to specific adsorption sites on an atomic scale. The rotational distribution of squares, rhombs, and the characteristic dodecagons of the NGT reveal a clear preference for an alignment of edges parallel to the 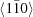 directions of the hexagonal substrate. This is a consequence of individual unit cells of an approximant structure. These approximant patches are locally reducing the symmetry in the 2D layer. Despite these local features, the dodecagonal symmetry remains on global scales as confirmed by the tiling element ratio and by diffraction. 

## Supplementary Material

Raw data for the STM image shown in Fig. 2(a). DOI: 10.1107/S2053273319000056/vf5004sup1.pdf

Raw data for Fig. 2(a) in tif format. DOI: 10.1107/S2053273319000056/vf5004sup2.tif
