# Supplementary material for: Full real-space analysis of a dodecagonal quasicrystal
Source: Acta Crystallogr A Found Adv. 2019 Feb 28;75(Pt 2):307–13. doi: 10.1107/S2053273319000056 (PMC6396402; doi:10.1107/S2053273319000056)

Acta Cryst A FOUNDATIONS ADVANCES

Volume 75 (2019)

Supporting information for article:

Full real-space analysis of a dodecagonal quasicrystal

Sebastian Schenk, Eva Maria Zollner, Oliver Krahn, Berit Schreck, René Hammer, Stefan Förster and Wolf Widdra

Raw data of the STM image shown in Fig. 2(a) upon subtraction of a polynomial background. The scale bar represents 10 nm. I = 15 pA, U = -1V, Δz = 600 pm.

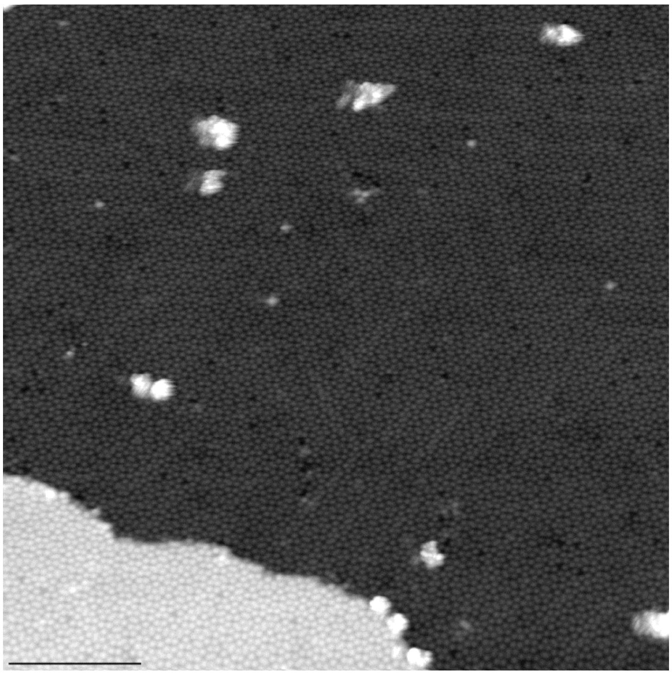

Supplement: Supplementary file 1 [file a-75-00307-sup1.pdf]
