# Supplementary figures and images for: Full real-space analysis of a dodecagonal quasicrystal
Source: Acta Crystallogr A Found Adv. 2019 Feb 28;75(Pt 2):307–13. doi: 10.1107/S2053273319000056 (PMC6396402; doi:10.1107/S2053273319000056)

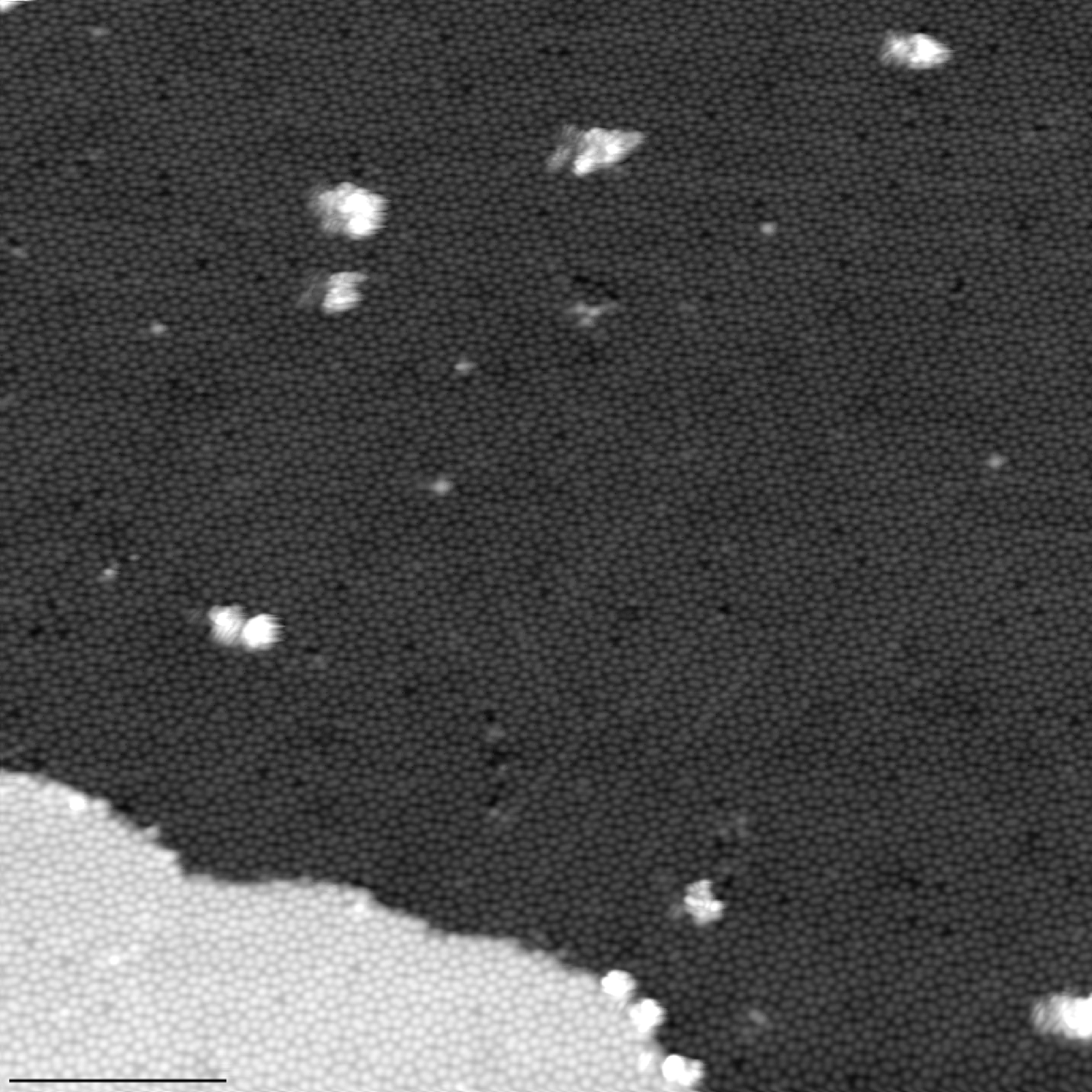

Supplement: Supplementary file 2 [file a-75-00307-sup2.tif]
